# Apical sparing of longitudinal strain in speckle-tracking echocardiography: A sensitive and specific finding in cardiac amyloidosis

**DOI:** 10.1007/s12471-018-1146-9

**Published:** 2018-08-14

**Authors:** J. Gil, L. Abreu, H. Antunes, M. L. Gonçalves, M. I. Pires, D. Moreira, E. Correia, L. S. Santos, J. C. Cabral

**Affiliations:** Cardiology Department, Centro Hospitalar Tondela-Viseu, Viseu, Portugal

**Fig. 1:**
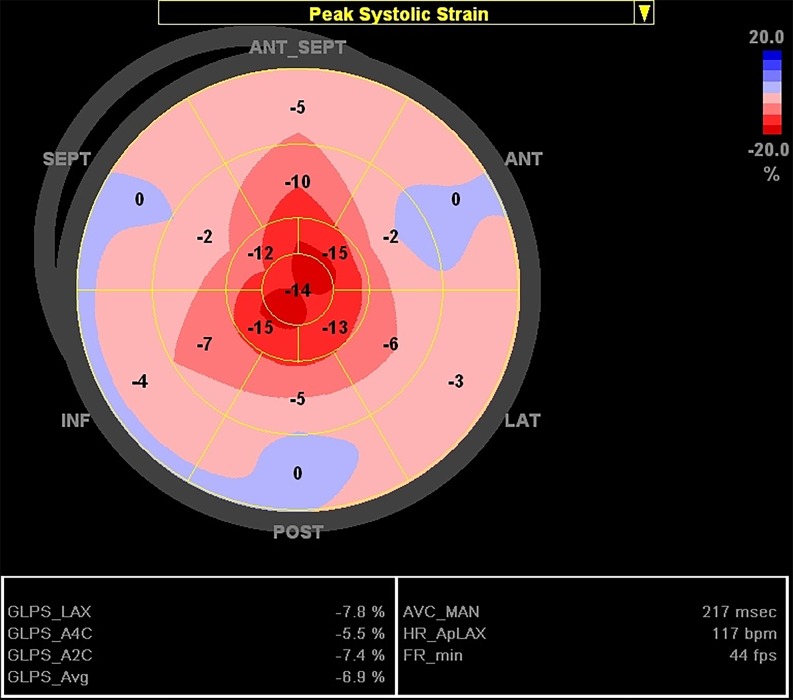
Apical sparing of longitudinal strain (LS) in cardiac amyloidosis. Global LS was of −6.9%. The mean absolute values of LS in the apex, mid-cavity and basal section of the left ventricle were −13.8, −5.3 and −2.0% respectively. Relative apical sparing is confirmed through the equation average apical LS/(average basal LS + mid-LS) [1], which in this patient resulted in a value of 1.89. A value of over 1.0 is highly sensitive and specific for diagnosing cardiac amyloidosis when compared with other causes of left ventricular hypertrophy [1]

A 47-year-old man was admitted for acute heart failure. The ECG had low voltage criteria. Transthoracic echocardiogram showed bi-atrial dilation, left ventricular concentric hypertrophy with reduced ejection fraction (LVEF of 48%) and hypertrophy of the right ventricular free wall. There was no valve dysfunction. Two-dimensional speckle-tracking echocardiography showed an apical sparing pattern of longitudinal strain (Fig. 1). Electrophoresis of serum proteins and bone marrow biopsy confirmed multiple myeloma. This suggested AL-type amyloidosis, and abdominal fat biopsy demonstrated amyloid-like, birefringent substance deposition in polarised light. Chemotherapy was started, remission occurred, and the patient remained stable after over 3 years of follow-up.

Amyloidosis is a multi-systemic disease characterised by the deposition of amyloid fibrils, cardiac involvement being usually associated with poor prognosis [1, 2]. Relative sparing of longitudinal strain in the left ventricular apex, which may be related to less amyloid deposition occurring in the apex than in the base, is highly sensitive and specific for the diagnosis of cardiac amyloidosis [1, 2].
